# Gender differences in the associations between body mass index, depression, anxiety, and stress among endocrinologists in China

**DOI:** 10.1186/s40359-023-01150-1

**Published:** 2023-04-14

**Authors:** Fei Xie, Licong Jiang, Yuanli Liu, Mingxiao Wang, Huanzhong Liu, Feng Jiang, Yinuo Wu, Yi-Lang Tang

**Affiliations:** 1 Department of Outpatient, Taihe Hospital, Hubei University of Medicine, Shiyan, Hubei China; 2 School of Medicine and Pharmacy, Wuhan University of Bioengineering, Wuhan, Hubei China; 3 School of Health Policy and Management, Chinese Academy of Medical Sciences & Peking Union Medical College, Beijing, China; 4 Department of Cardiology, Emergency General Hospital, Beijing, China; 5 Department of Psychiatry, Chaohu Hospital of Anhui Medical University, Hefei, China; 6 Department of Psychiatry, School of Mental Health and Psychological Sciences, Anhui Medical University, Hefei, China; 7 Anhui Psychiatric Center, Hefei, China; 8 School of International and Public Affairs, Shanghai Jiao Tong University, Shanghai, China; 9 Institute of Healthy Yangtze River Delta, Shanghai Jiao Tong University, Shanghai, China; 10 Chinese Academy of Medical Sciences & Peking Union Medical College, Beijing, China; 11 Department of Psychiatry and Behavioral Sciences, Emory University, Atlanta, GA USA; 12 Atlanta VA Medical Center, Decatur, GA USA

**Keywords:** Endocrinologists, Body mass index, Overweight, Mental health, Gender difference

## Abstract

**Background:**

Depression, anxiety, and stress symptoms have been found to be associated with overweight or obesity, but the gender differences in the associations have not been well-examined. Based on a national sample of endocrinologists in China, we examined such associations with a focus on gender differences.

**Methods:**

Data were collected from endocrinologists in China using an online questionnaire, which included demographic data, body weight, and height. Depression, anxiety, and stress symptoms were assessed using the Depression, Anxiety, and Stress Scale-21 (DASS-21).

**Results:**

In total, 679 endocrinologists (174 males and 505 females) completed the survey. One-fourth (25.6%) were classified as overweight, with a significant gender difference (48.9% in males vs. 17.6% in females, *p* < 0.05). Overall, 43.4% of the participants endorsed probable depressive symptoms (54.6% in males and 39.6% in females, *p* = 0.004), 47.6% for anxiety (51.7% in males vs. 46.1% in females, *p* = 0.203), and 29.6% for stress symptoms (34.5% in males vs. 27.92% in females, *p* = 0.102). After controlling for confounders, in the whole group, male gender (aOR = 4.07, 95% CI:2.70–6.14, *p* < 0.001), depression (aOR = 1.05, 95% CI:1.00-1.10, *p* = 0.034) and age (aOR = 1.03, 95% CI:1.00-1.05, *p* = 0.018) were positively associated with overweight. In males, depression (aOR = 1.14, 95% CI:1.05–1.25, *p* = 0.002), administration position (aOR = 4.36, 95% CI:1.69–11.24, *p* = 0.002), and night shifts/month (aOR = 1.26, 95% CI:1.06–1.49, *p* = 0.008) were positively associated with overweight, while anxiety (aOR = 0.90, 95% CI:0.82–0.98, *p* = 0.020) was negatively associated with overweight. In females, only age (aOR = 1.04, 95% CI:1.01–1.07, *p* = 0.014) was significantly associated with overweight status, while depression and anxiety were not associated with overweight. Stress symptoms were not associated with overweight in either gender.

**Conclusions:**

One-fourth of endocrinologists in China are overweight, with a rate in males nearly triple the one in females. Depression and anxiety are significantly associated with overweight in males but not females. This suggests possible differences in the mechanism. Our findings also highlight the need to screen depression and overweight in male physicians and the importance of developing gender-specific interventions.

**Supplementary Information:**

The online version contains supplementary material available at 10.1186/s40359-023-01150-1.

## Introduction

Overweight and obesity, abnormal or excessive fat accumulation in the body, have become a worsening public health concern in China [1]. In recent years, the prevalence of overweight increased rapidly, parallel to Chinese social, economic, and environmental transitions [2]. According to a recent national survey, more than half of Chinese adults were overweight or obese [3]. The increase in overweight and obesity is believed to impact the burden of non-communicable diseases, such as type 2 diabetes, cardiovascular disease, and cancer [4–6].

Overweight and obesity are often multifactorial, including factors contributing to gaining and retaining excess weight. Commonly mentioned factors include social and environmental factors (such as fast food culture, sedentary work environment, etc.), and personal factors (such as diet, lack of exercise, genetics, and mental health symptoms) [7–12].

Mental health symptoms and body weight may interact in several manners. First, mental health symptoms may lead to changes in eating habits and levels of physical activity [13, 14]. Depression, anxiety, and stress can disrupt normal eating habits and lead to overeating or loss of appetite, both of which can contribute to weight gain [15]. Second, overweight or obesity can lead to mental health symptoms. Being overweight or obese can lead to low self-esteem, body dissatisfaction, and depression [16]. Overweight or obesity can also lead to medical problems that can cause pain, limited mobility, and decreased quality of life, all of which can contribute to anxiety and stress [17]. Third, mental health problems and overweight share some common risk factors, such as genetics [18], biological mechanisms [19], and environmental factors [20]. For example, high-calorie foods, lack of physical activity, and high levels of stress can contribute to both mental health symptoms and overweight/obesity [21].

With respect to the relationship between mental health symptoms and overweight/obesity, gender may be a potential moderating factor [22]. On one hand, males and females have different physiological responses to stress, with men being more likely to overeat and women being more likely to lose their appetite. Women may be more likely to cope with depression through emotional eating, while men may be more likely to cope through binge drinking or other behaviors. These differences may be due to differences in hormonal responses to depression, as cortisol is a key intermediate factor in this relationship [23, 24]. On the other hand, there are often different cultural and societal expectations for men and women. Women are often expected to conform to a thinner body image, which can contribute to increased levels of body dissatisfaction, anxiety, and depression. In contrast, men are often expected to be strong and muscular, which can lead to increased pressure to maintain a larger body size [25, 26].

The gender differences in the relationship between mental health symptoms and body weight highlight the importance of including gender in health research and interventions. Interventions tailored to gender-specific challenges may improve healthy body weight and mental health. Such interventions may also better address the unique challenges faced by each gender.

Due to the high prevalence of diabetes and other metabolic disorders, endocrinologists play an important and unique role in the Chinese healthcare system. For example, diabetes is a major public health challenge in China with a prevalence of 9.7% or over 114 million people living with the disease in 2017 [27]. This statistic is estimated to rise to over 131 million people living with the disease by 2040 [28]. Thyroid disorders are also a major health concern in mainland China, where a significant portion of the population is affected by thyroid nodules and subclinical hypothyroidism. The prevalence of thyroid nodules in China is reported to be 20.43%, while subclinical hypothyroidism affects 12.93% of the population [29]. Endocrinologists are medical specialists who have the necessary training and expertise to diagnose, treat and manage diabetes and other endocrine disorders. Endocrinologists often provide comprehensive care to patients with diabetes, including medical management, lifestyle modifications, and ongoing monitoring of diabetes-related complications. Endocrinologists are also equipped to manage other metabolic disorders, such as obesity, which is a major risk factor for diabetes in China. Through their specialized training, they can provide effective interventions to help prevent the development of obesity and related conditions [30].

While they may have a better awareness of the importance of keeping a healthy weight and have more professional knowledge of healthy diets and lifestyles [31], they may also experience mental health symptoms, owing to stress related to heavy caseloads, long working hours, and difficulty maintaining work-life balance. To date, the prevalence of overweight and obesity and their correlates in Chinese endocrinologists have not been examined, especially in a large, national sample. Additionally, the relationships between overweight and mental health symptoms are of interest, as they may shed light on interventions.

In this study, based on a nationwide survey of practicing endocrinologists, we aimed to examine: (1) the prevalence of overweight and obesity and their correlates, (2) the prevalence of probable depressive, anxiety, and stress symptoms, (3) the associations between overweight and mental health symptoms, and (4) gender differences in the associations. We hypothesize that: (1) there is a significant association between obesity and depression among women, but not in men, and (2) there is a significant association between obesity and anxiety/stress in both genders.

## Materials and methods

### Study design and participants

The study was conducted between March 18th and 31st, 2019. It was part of the China Healthcare Improvement Initiative (CHII) supported by the National Health Committee [32]. The goal of CHII was to improve the health and well-being of medical staff and healthcare quality in China’s province-level top-tier general hospitals. The Provincial People’s Hospitals of each province in mainland China were purposely selected by CHII, as they are the top-tier hospitals in the region. In the Chinese healthcare system, each province has one Provincial People’s Hospital and it often has the best resources in budget, equipment, and staffing. It also usually serves as a tertiary referral center for the province. We recruited 31 hospitals, which are all government-owned, province-level, general tertiary hospitals. They accounted for 1.85% of the beds of all tertiary hospitals and 2.68% of all patient care among tertiary hospitals in mainland China [33]. All endocrinologists working in those hospitals were invited to participate in an anonymous online survey, which was delivered using WeChat, a popular Social App in China. To avoid duplicate responses, each cell phone could only submit responses once.

The survey protocol was reviewed and approved by the Ethics Committee of Emergency General Hospital in Beijing. Each participant had to complete the consent form before they could proceed to the survey.

### Measures

The basic sociodemographic features and work-related information were collected based on a literature review [34–36]. They included age, gender, number of children, education, relationship status, height, weight, cigarette smoker or not, professional title, administration position, sleep hours/day, work hours/week, and night shifts/month.

Body mass index (BMI) was calculated using the participants’ self-reported height and weight. According to the Working Group on Obesity in China, the cutoff values for BMI (unit: kg/m²) in Chinese adults are: underweight: <18.5; normal: 18.5–24.0; overweight but not obesity: 24.0 ≤ BMI<30.0; obesity:≥30.0 [1, 2, 37].

Mental health symptoms were assessed using the Chinese version of the Depression, Anxiety, and Stress Scales-21 (DASS-21) [38, 39]. DASS-21 is a 21-item self-report questionnaire to investigate three symptom domains, including depressive, anxious, and stress symptoms. The instrument uses a 4-point Likert scale. Each item was scored from 0 (did not apply to me at all over the last week) to 3 (applied to me very much over the past week). As the DASS-21 is a short-form version of the Long Form DASS, the final score of each item was multiplied by two. The level of symptoms was ranked as normal, mild, moderate, severe, and extremely severe, according to certain cut-off values [38]. The Cronbach’s alpha of DASS-21 was 0.96 in this study.

### Data analysis

The normality of research data was detected through a one-sample K-S test. Quantitative variables were expressed as mean (SD) or median (IQR), and qualitative variables were expressed as frequencies (percentages). The mean/median scores and prevalence rates of depression, anxiety, and stress were calculated.

Depression, anxiety, and stress scores were compared using the Mann-Whitney test or Kruskal-Wallis test among different BMI groups and different gender groups, as appropriate. The gender and BMI groups interactions were also tested in the same process with a newly generated variable of gender×BMI groups. Depression, anxiety, and stress prevalence rates were compared using Pearson’s χ^2^ test or Fisher’s exact test, among different BMI groups and different gender groups.

We used the Pearson correlation analysis to test the correlation between depression, anxiety, stress scores, and continuous BMI values between males and females, respectively.

We performed separate multilevel binary logistic regression analyses with endocrinologists in level 1 and hospitals in level 2. Only the overweight and normal BMI groups (reference group) were involved in the logistic regression, and the weight status was treated as the dependent variable. Depression, anxiety, and stress scores were treated as continuous independent variables and were purposely included in the regression, while other confounders were screened through backward stepwise regression. We performed the regression models in entire groups, male and female subgroups, respectively.

We performed all analyses using the STATA software version 16.0 (Stata Corporation, College Station, TX, USA). Two-sided *p* < 0.05 was considered significant.

## Results

### Sample characteristics

At the beginning of the survey, 879 endocrinologists were invited to participate in this survey, and 711 endocrinologists responded (response rate = 80.9%). Finally, 679 endocrinologists completed the questionnaire without logical errors. Table 1 shows their detailed sociodemographic features, job-related factors, and gender differences.

**Table 1:** Basic Characteristics of 679 endocrinologists in China

Characteristic		N (%)	Male (174)	Female (505)	*p*
Relationship status (N, %)					0.841
	Not married	73(10.75)	18(10.34)	55(10.89)	
	Married	606(89.25)	156(89.66)	450(89.11)	
Children (N, %)					**0.004**
	None	145(21.35)	29(16.67)	116(22.97)	
	One	432(63.62)	106(60.92)	326(64.55)	
	Two or more	102(15.02)	39(22.41)	63(12.48)	
Educational level* (N, %)					
	Medical/college degree	118(17.38)	34(19.54)	84(16.63)	0.221
	Add on a Master’s degree	339(49.93)	77(44.25)	262(51.88)	
	Add on a Doctoral degree	222(32.70)	63(36.21)	159(31.49)	
Professional title (N, %)					**0.003**
	Junior	113(16.64)	19(10.92)	94(18.61)	
	Middle	241(35.49)	53(30.46)	188(37.23)	
	Senior	325(47.86)	102(58.62)	223(44.16)	
Administration position (N, %)					
	No	580(85.42)	133(76.44)	447(88.51)	**< 0.001**
	Yes	99(14.58)	41(23.56)	58(11.49)	
Cigarette Smoker (N, %)					**< 0.001**
	No	654(96.32)	149(85.63)	505(100.00)	
	Yes	25(3.68)	25(14.37)	0(0)	
BMI category (N, %)					**< 0.001**
	Underweight (BMI < 18.5 kg/m^2^)	38(5.60)	2(1.15)	36(7.13)	
	Normal (18.5 kg/m^2^ ≤ BMI < 24.0 kg/m^2^)	467(68.78)	87(50.00)	380(75.25)	
	Overweight (24 kg/m^2^ ≤ BMI)	174(25.63)	85(48.85)	89(17.62)	
		Mean (SD)	Mean (SD)	Mean (SD)	*p*
Age (years)		39.59(8.48)	42.56(8.48)	38.57(8.25)	**< 0.001**
Sleep hours /day		6.35(0.76)	6.41(0.72)	6.33(0.77)	0.226
Work hours/week		55.08(13.98)	54.65(13.49)	55.22(14.15)	0.645
BMI		22.30(2.64)	23.91(2.45)	21.74(2.46)	**< 0.001**
		Median (IQR)	Median (IQR)	Median (IQR)	*p*
Night shifts/month		4(3)	4(3)	4(2.5)	0.124

*In China, medical school graduates are awarded a bachelor’s degree in medicine (similar to the European system). Some obtain a master’s or doctoral degree in addition to their medical degree. Bold value for *p* < 0.05.

Categorical variables are reported as N (%). Continuous variables (age, sleep hours, work hours, and BMI) were reported as Mean (SD) unless indicated otherwise. Based on the Chinese criteria, 24.0% of the endocrinologists were classified as overweight but not obese, 1.62% were obese. As the sample of obese participants was small, we combined the two groups as “overweight” in our analysis unless specified otherwise.

After merging the overweight and obese groups, about one-fourth (25.6%) were overweight, 48.85% in males, 17.6% in females; 5.6% underweight, 1.2% in males, and 7.1% in females. A significant gender difference was found in BMI categories (*p* < 0.001). Nearly one-quarter (24.7%) of overweight endocrinologists were less than 35 years old, 39.7% were between 35 and 44 years old, and 35.6% were in the group of 45 years or older.

In this sample, we found the overall prevalence of probable depression was 43.4%, with a significant gender difference (54.6% in males and 39.6% in females, *p* = 0.004). We also found a significant gender difference (*p* = 0.032) and gender×BMI category interaction for depression scores (*p* = 0.035).

Nearly one-half (47.6%) of endocrinologists endorsed probable anxious symptoms, 51.7% in males and 46.1% in females (*p* = 0.203), respectively. We did not find a significant gender difference (*p* = 0.549) nor gender×BMI category interaction for anxiety scores (*p* = 0.615).

The rate of probable stress symptoms was 29.6% in this sample, 34.5% in males, and 27.92% in females (*p* = 0.102), respectively. There was a gender difference (*p* = 0.036) in stress scores, but no gender×BMI category interaction for anxiety scores (*p* = 0.087) (see Table 2).

**Table 2:** DASS scores in male and female endocrinologists in China

	Total (N = 679)	Male (N = 174)				Female (N = 505)			Gender χ^2^ (*p* value)	BMI category χ^2^ (*p* value)	Gender*BMI category χ^2^ (*p* value)
		Underweight (N = 2)	Normal (N = 87)	Overweight (N = 85)		Underweight (N = 36)	Normal (N = 380)	Overweight (N = 89)			
Depression score	8(12)	7(14)	8(10)	12(12)		7(11)	8(12)	6(12)	2.14(**0.032**)	3.83(0.147)	11.95(**0.035**)
Anxiety score	6(10)	9(18)	6(10)	8(12)		8(14)	6(10)	6(10)	0.60(0.549)	1.76(0.415)	3.56(0.615)
Stress score	12(10)	8(16)	12(14)	14(8)		12(10)	12(10)	10(12)	2.10(**0.036**)	0.83(0.662)	9.60(0.087)

Bold value for *p* < 0.05. The numbers in the table are the DASS-21 subscale scores, and the numbers in the parenthesis are the interquartile range.

We performed Pearson correlations between depression, anxiety, stress scores, and continuous BMI values, but did not find significant correlations (Supplemental Table 1).

Supplemental Table 2 shows detailed information on depression, anxiety, and stress symptoms in categories.

We then performed stepwise multilevel logistic regression analysis, using overweight status (overweight + obesity) as a dependent variable, and others as independent variables. Depression, anxiety, and stress scores were purposely maintained. We performed the analysis in the whole sample, and male and female participants, separately.

In the whole group, male gender (aOR = 4.07, 95% CI:2.70–6.14, *p* < 0.001), depression scores (aOR = 1.05, 95% CI:1.00-1.10, *p* = 0.034) and age (aOR = 1.03, 95% CI:1.00-1.05, *p* = 0.018) were positively associated with overweight status.

In the male group, depression scores (aOR = 1.14, 95% CI:1.05–1.25, *p* = 0.002), administration position (aOR = 4.36, 95% CI:1.69–11.24, *p* = 0.002), and night shifts/month (aOR = 1.26, 95% CI:1.06–1.49, *p* = 0.008), were positively associated with overweight status, while anxiety scores (aOR = 0.90, 95% CI:0.82–0.98, *p* = 0.020) were negatively associated with overweight status. Stress scores were not associated with overweight status in males.

In the female group, only age (aOR = 1.04, 95% CI:1.01–1.07, *p* = 0.014) was positively associated with overweight status. While depression, anxiety, and stress scores were not associated with overweight status. For details, please see Table 3.

**Table 3:** Gender differences in the association between DASS scores and overweight status in male and female endocrinologists

Variable	All (N = 679)				Male (N = 174)				Female (N = 505)				
	aOR	95% CI (Lower)	95% CI (Upper)	*p*	aOR	95% CI (Lower)	95% CI (Upper)	*p*	aOR	95% CI (Lower)	95% CI (Upper)	*p*	
Gender (ref. Female)	4.07	2.70	6.14	**< 0.001**	-	-	-	-	-	-	-	-	
Depression	1.05	1.00	1.10	**0.034**	1.14	1.05	1.25	**0.002**	1.01	0.95	1.07	0.730	
Anxiety	0.98	0.93	1.03	0.474	0.90	0.82	0.98	**0.020**	1.01	0.94	1.08	0.796	
Stress	0.98	0.94	1.03	0.394	1.00	0.92	1.07	0.915	0.97	0.91	1.03	0.304	
Age	1.03	1.00	1.05	**0.018**	-	-	-	-	1.04	1.01	1.07	**0.014**	
Administration position (ref. No)	-	-	-	**-**	4.36	1.69	11.24	**0.002**	-	-	-	-	
Night shifts/month	-	-	-	-	1.26	1.06	1.49	**0.008**	-	-	-	-	

Bold value for *p* < 0.05. – indicates the variable was not included in the final model of the stepwise regression.

In the sensitivity analysis, we excluded the participants who were classified as obese (5 males and 6 females), and the overall findings remained largely unchanged compared with the analysis which included obese participants (see supplemental Table 3).

## Discussion

This research was one of the first to focus on overweight and mental health among Chinese physicians. Our main findings include: (1) one-fourth of endocrinologists were overweight, nearly half of the participants endorsed depression or anxiety, and one-third endorsed feeling stressed; (2) overweight was significantly associated with depression and anxiety scores in male endocrinologists, but not in females; (3) stress scores were not significantly associated with overweight in males or females. Our findings suggest that gender might moderate the relationship between depression/anxiety and body weight.

In this survey, the prevalence of overweight/obese among endocrinologists was 25.6% (1.62% for obese only). This figure is lower than the average level of Chinese adults, as the China Chronic Disease and Nutrition Surveillance 2015-19 survey showed that the prevalence for overweight and obesity was 34.3% and 16.4% in people aged 18 years and older [1]. This encouraging finding may be related to better awareness and body weight management skills in this population [31].

Our findings show that a significant association between overweight status and depression was only found in males (not in females), which is contrary to our hypothesis but is consistent with one prior study, a survey involving German and Chinese participants [40]. Another study showed that a lack of positive emotion was associated with being overweight in females, whereas significant associations were found between all dimensions of depression and overweight in males [36].

Similar to the association between overweight and depression, we also found that the association between overweight/obesity and anxiety was only significant in males, not in females, which partially aligned with the research hypothesis. This is in line with the findings of an international study. In their study of 9,007 Chinese university students and 364 German students, Lavallee et al. only found significant associations in Chinese male students between overweight and mental health symptoms, as assessed using DASS-21 [40]. Other studies have reported no gender-specific association between overweight and anxiety [41–43], while one reported the association only in female empty nesters [44]. Therefore, this interesting finding needs further research.

The finding that stress was not significantly associated with overweight in both genders was not consistent with our hypothesis. The relationships between stress and obesity in susceptible individuals can be partly traced back to a third key player: increased glucocorticoid (GC) action, which is influenced by individual GC sensitivity [45]. Multiple genetic and disease-related factors can influence GC sensitivity with variations among different populations [46]. The biological characteristics of Chinese endocrinologists might be the potential factors for lack of the association.

We found that depression and anxiety may contribute to being overweight in males but not in females, which has theoretical and held practical implications for the understanding of the complex relationship between mental health and body weight. Gender-specific pathways may moderate the relationship between mental health and body weight. Identifying these pathways could lead to gender-specific interventions which may help prevent and treat overweight. These findings highlight the importance of considering gender when evaluating and treating overweight, as well as addressing depression and anxiety symptoms. Healthcare providers should take a gender-specific approach when working with patients to manage weight, considering the potential role of mental health in the development and maintenance of overweight. The results of this research have implications for public health as well as the role of gender-specific factors, including the role of depression and anxiety, which should be considered when developing interventions for overweight or obesity.

The present study has a few limitations. First, the findings were based on a cross-sectional survey, and it is difficult to infer causality between different variables. Second, the survey data may have recall bias, as collected through self-report. Third, the sample was only from 31 tertiary public hospitals in China, so the generalizability of the study conclusions may be limited. Fourth, mental health was measured by questionnaires, and no clinical diagnoses can be provided. Finally, some important information related to BMI and mental health, such as health status and other lifestyle information (such as diet, exercise, etc.) were not collected.

## Conclusions

In a large national sample, we found that one-fourth of endocrinologists were overweight, which is lower than the data in the general population. Depression and anxiety may be significant contributors to overweight in males, but not in females, which suggests the mechanism of the association between overweight and mental health in males and females are different. Further studies are needed to identify mechanisms that lead to more gender-dependent screening and therapies. Different interventional strategies may also be needed when addressing overweight issues in male and female physicians.

## Electronic supplementary material

Below is the link to the electronic supplementary material.

Supplementary Material 1

## Data Availability

The datasets generated and analyzed for the current study are not publicly available due to the necessity to ensure participant confidentiality policies and laws of the country. But they are available from the corresponding author upon reasonable request.
