# Supplementary material for: Gender differences in the associations between body mass index, depression, anxiety, and stress among endocrinologists in China
Source: BMC Psychol. 2023 Apr 14;11:116. doi: 10.1186/s40359-023-01150-1 (PMC10103496; doi:10.1186/s40359-023-01150-1)
Supplement: Supplementary file 1 — Supplementary Material 1 [file 40359_2023_1150_MOESM1_ESM.docx]

Supplemental Table 1. Pearson correlations between DASS scores and BMI in 679 endocrinologists in China

|  | Group | *r* | *p* value |
| --- | --- | --- | --- |
| Depression | All | 0.03 | 0.500 |
|  | Male | 0.08 | 0.282 |
|  | Female | -0.02 | 0.592 |
| Anxiety | All | -0.05 | 0.192 |
|  | Male | -0.06 | 0.423 |
|  | Female | -0.07 | 0.139 |
| Stress | All | -0.01 | 0.762 |
|  | Male | 0.02 | 0.783 |
|  | Female | -0.06 | 0.198 |

Supplemental Table 2. DASS rank in male and female endocrinologists in China (%)

|  | | Total  (N=679) | Male (N=174) | | |  | Female (N=505) | | | Gender  χ^2^  (*p* value) | BMI category  χ^2^  (*p* value) |
| --- | --- | --- | --- | --- | --- | --- | --- | --- | --- | --- | --- |
|  |  |  | Underweight  (N=2) | Normal  (N=87) | Overweight  (N=85) |  | Underweight  (N=36) | Normal  (N=380) | Overweight  (N=89) |  |  |
| Depression | |  |  |  |  |  |  |  |  |  |  |
|  | Normal | 384(56.55) | 1(50.00) | 48(55.17) | 30(35.29) |  | 23(63.89) | 227(59.74) | 55(61.80) | 14.51  (**0.006**) | 13.27  (0.117) |
|  | Mild | 112(16.49) | 0 | 19(21.84) | 21(24.71) |  | 5(13.89) | 59(15.53) | 8(8.99) |  |  |
|  | Moderate | 137(20.18) | 1(50.00) | 15(17.24) | 28(32.94) |  | 5(13.89) | 67(17.63) | 21(23.60) |  |  |
|  | Severe | 20(2.95) | 0 | 3(3.45) | 1(1.18) |  | 0 | 12(3.16) | 4(4.49) |  |  |
|  | Extremely severe | 26(3.83) | 0 | 2(2.30) | 5(5.88) |  | 3(8.33) | 15(3.95) | 1(1.12) |  |  |
| Anxiety | |  |  |  |  |  |  |  |  |  |  |
|  | Normal | 356(52.43) | 1(50.00) | 47(54.02) | 36(42.35) |  | 16(44.44) | 203(53.42) | 53(59.55) | 8.24  (0.083) | 10.85  (0.245) |
|  | Mild | 58(8.54) | 0 | 6(6.90) | 9(10.59) |  | 3(8.33) | 35(9.21) | 5(5.62) |  |  |
|  | Moderate | 159(23.42) | 0 | 23(26.44) | 29(34.12) |  | 6(16.67) | 86(22.63) | 15(16.85) |  |  |
|  | Severe | 53(7.81) | 1(50.00) | 3(3.45) | 4(4.71) |  | 6(16.67) | 27(7.11) | 12(13.48) |  |  |
|  | Extremely severe | 53(7.81) | 0 | 8(9.20) | 7(8.24) |  | 5(13.89) | 29(7.63) | 4(4.49) |  |  |
| Stress | |  |  |  |  |  |  |  |  |  |  |
|  | Normal | 478(70.40) | 1(50.00) | 61(70.11) | 52(61.18) |  | 21(58.33) | 277(72.89) | 66(74.16) | 2.84  (0.533) | 11.25  (0.226) |
|  | Mild | 90(13.25) | 1(50.00) | 12(13.79) | 15(17.65) |  | 9(25.00) | 42(11.05) | 11(12.36) |  |  |
|  | Moderate | 58(8.54) | 0 | 6(6.90) | 11(12.94) |  | 2(5.56) | 34(8.95) | 5(5.62) |  |  |
|  | Severe | 36(5.30) | 0 | 6(6.90) | 4(4.71) |  | 4(11.11) | 17(4.47) | 5(5.62) |  |  |
|  | Extremely severe | 17(2.50) | 0 | 2(2.30) | 3(3.53) |  | 0 | 10(2.63) | 2(2.25) |  |  |

Bold value for *p*<0.05

Supplemental Table 3. Sensitivity analysis of gender differences in the association between DASS scores and overweight status after excluding participants with obesity

| Variable | ALL (N=679) | | | |  | Male (N=174) | | | |  | Female (N=505) | | | |
| --- | --- | --- | --- | --- | --- | --- | --- | --- | --- | --- | --- | --- | --- | --- |
|  | aOR | 95% CI (Lower) | 95% CI (Upper) | *p* |  | aOR | 95% CI (Lower) | 95% CI (Upper) | *p* |  | aOR | 95% CI (Lower) | 95% CI (Upper) | *p* |
| Gender (ref. Female) | 4.00 | 2.65 | 6.04 | **<0.001** |  | - | - | - | - |  | - | - | - | - |
| Depression | 1.05 | 1.00 | 1.10 | **0.037** |  | 1.15 | 1.06 | 1.26 | **0.002** |  | 1.01 | 0.95 | 1.07 | 0.769 |
| Anxiety | 0.98 | 0.93 | 1.03 | 0.508 |  | 0.91 | 0.83 | 1.00 | **0.042** |  | 1.01 | 0.94 | 1.08 | 0.816 |
| Stress | 0.98 | 0.93 | 1.03 | 0.379 |  | 0.98 | 0.91 | 1.06 | 0.589 |  | 0.97 | 0.91 | 1.03 | 0.341 |
| Age | 1.03 | 1.00 | 1.05 | **0.027** |  | - | - | - | - |  | 1.03 | 1.01 | 1.05 | **0.019** |
| Administration position (ref. No) | - | - | - | **-** |  | 4.85 | 1.82 | 12.93 | **0.002** |  | - | - | - | - |
| Night shifts/month | - | - | - | - |  | 1.28 | 1.07 | 1.52 | **0.006** |  | - | - | - | - |

Bold value for *p*<0.05. – indicates the variable was not included in the final model of the stepwise regression
